# PI3K/Akt1 Pathway Suppression by Quercetin–Doxorubicin Combination in Osteosarcoma Cell Line (MG-63 Cells)

**DOI:** 10.3390/medicina61081347

**Published:** 2025-07-25

**Authors:** Mehmet Uğur Karabat, Mehmet Cudi Tuncer

**Affiliations:** 1 Department of Histology and Embryology, Medical Faculty, Dicle University, Diyarbakır 21280, Turkey; ugurkrbt@hotmail.com; 2 Department of Anatomy, Faculty of Medicine, Dicle University, Diyarbakır 21280, Turkey

**Keywords:** quercetin, osteosarcoma, PI3K/Akt signaling pathway, proliferation, apoptosis

## Abstract

*Background and Objectives*: This study aimed to investigate the anticancer effects and potential synergistic interactions of quercetin (Q) and doxorubicin (Dox) on the MG-63 osteosarcoma (OS) cell line. Specifically, the effects of these agents on cell viability, apoptosis, reactive oxygen species (ROS) generation, antioxidant defense, and the phosphoinositide 3-kinase/protein kinase B (PI3K/Akt1) signaling pathway were evaluated. *Material and Methods*: MG-63 cells were cultured and treated with varying concentrations of Q and Dox, both individually and in combination (fixed 5:1 molar ratio), for 48 h. Cell viability was assessed using an MTT assay, and IC_50_ values were calculated. Synergistic effects were analyzed using the Chou–Talalay combination index (CI). Apoptosis was evaluated via Annexin V-FITC/PI staining and caspase-3/7 activity. ROS levels were quantified using DCFH-DA probe, and antioxidant enzymes (SOD, GPx) were measured spectrophotometrically. Gene expression (Runx2, PI3K, Akt1, caspase-3) was analyzed by reverse transcription quantitative polymerase chain reaction (RT-qPCR). *Results*: Q and Dox reduced cell viability in a dose-dependent manner, with IC_50_ values of 70.3 µM and 1.14 µM, respectively. The combination treatment exhibited synergistic cytotoxicity (CI < 1), especially in the Q50 + Dox5 group (CI = 0.23). Apoptosis was significantly enhanced in the combination group, evidenced by increased Annexin V positivity and caspase-3 activation. ROS levels were markedly elevated, while antioxidant enzyme activities declined. RT-qPCR revealed upregulation of caspase-3 and downregulation of Runx2, PI3K, and Akt1 mRNA levels. *Conclusions*: The combination of Q and Dox exerts synergistic anticancer effects in MG-63 OS cells by inducing apoptosis, elevating oxidative stress, suppressing antioxidant defense, and inhibiting the PI3K/Akt1 signaling pathway and Runx2 expression. These findings support the potential utility of Q as an adjuvant to enhance Dox efficacy in OS treatment.

## 1. Introduction

OS is the most common and aggressive primary malignant bone tumor, predominantly affecting children and adolescents, and is characterized by a high metastatic potential. Current treatment protocols typically involve a combination of neoadjuvant chemotherapy and surgical resection. Despite advances in therapeutic strategies, the 5-year survival rate remains at 60–70% for non-metastatic cases and falls below 20% in those with metastasis, underscoring the urgent need for the development of novel therapeutic approaches [1,2,3].

The standard treatment for OS involves high-dose chemotherapy administered before and after surgery, commonly using agents such as methotrexate, Dox, and ifosfamide [4]. Dox, an anthracycline chemotherapeutic agent, exerts its antitumor activity by inhibiting DNA topoisomerase II, thereby disrupting DNA replication and transcription. Moreover, it induces oxidative stress through the generation of free radicals, ultimately triggering apoptosis [5]. However, the clinical application of Dox is limited by its systemic toxicities—particularly cardiotoxicity—as well as the development of drug resistance over time [5,6].

In this context, the anticancer potential of natural compounds has garnered increasing attention. Numerous studies have demonstrated that plant-derived flavonoids possess chemopreventive and chemotherapeutic properties, with the ability to target cancer cells either alone or in combination with conventional chemotherapeutic agents [7]. Q (3,3′,4′,5,7-pentahydroxyflavone) is a naturally occurring flavonoid found in various dietary sources, including fruits, vegetables, onions, apples, and tea. Its anti-inflammatory, antioxidant, and antiproliferative effects have been extensively demonstrated in both in vitro and in vivo models [7,8]. Q has been shown to induce apoptosis, arrest the cell cycle, suppress metastasis, and modulate the intracellular redox balance by reducing oxidative stress. Although its efficacy has been established in multiple cancer types—including breast, prostate, colon, and lung cancers—its molecular effects on OS cells remain incompletely understood [8,9].

The phosphoinositide 3-kinase/protein kinase B (PI3K/Akt) signaling pathway plays a central role in the pathogenesis of OS, promoting cell proliferation, survival, and metastasis. Inhibition of this pathway has been shown to suppress tumor progression [10]. In addition, Runt-related transcription factor 2 (Runx2) is a key regulator of osteogenic differentiation and tumor invasion in OS [11]. Accordingly, agents that modulate PI3K/Akt signaling and Runx2 expression are considered promising therapeutic candidates.

**Figure 1:**
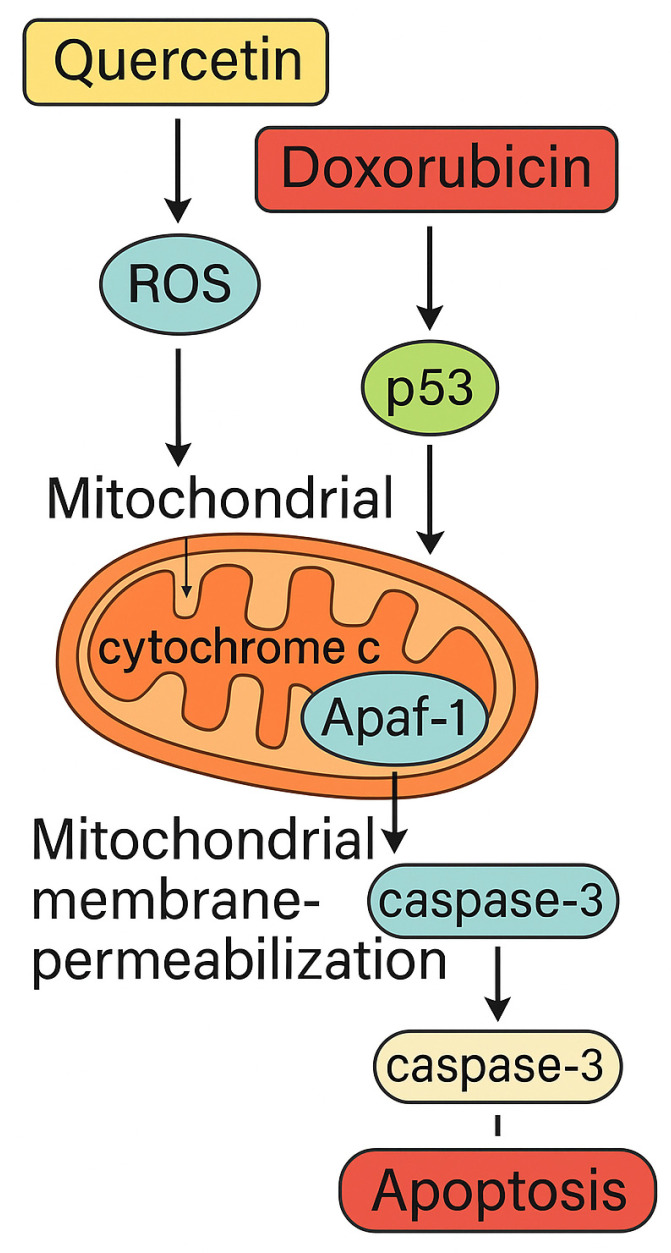
Schematic representation of the molecular pathways involved in the synergistic apoptotic effect of quercetin and doxorubicin in MG-63 osteosarcoma cells. This diagram illustrates how Q and Dox collaboratively induce oxidative stress and mitochondrial-mediated apoptosis. Quercetin increases intracellular ROS, leading to mitochondrial dysfunction and cytochrome c release. This promotes apoptosome formation through Apaf-1 and triggers the caspase cascade via caspase-9 and caspase-3, culminating in apoptosis. Doxorubicin contributes to this process by activating the p53 pathway, which amplifies the apoptotic signal. This Figure summarizes the key molecular interactions confirmed in this study through qPCR, ROS assays, and caspase activity analysis. Apaf-1: Apoptotic Protease Activating Factor 1; p53: Tumor Protein p53; ROS: reactive oxygen species.

In this study, we aimed to elucidate the potential apoptotic mechanisms underlying the effects of Q and Dox in MG-63 OS cells. Both compounds are known to influence distinct molecular targets that ultimately converge on common apoptotic pathways. Q exerts its pro-apoptotic effect primarily by increasing intracellular ROS and disrupting mitochondrial membrane potential, whereas Dox induces DNA damage and activates the p53 signaling cascade. These upstream molecular events are closely associated with the intrinsic mitochondrial apoptotic pathway, characterized by cytochrome c release, apoptosome assembly through Apaf-1 and caspase-9 activation, and downstream execution by caspase-3. The proposed model summarizing these interacting apoptotic cascades induced by Q and Dox is illustrated in Figure 1.

The present study aimed to evaluate the cytotoxic, apoptotic, and antiproliferative effects of Q and Dox on the MG-63 OS cell line. Specifically, we investigated their effects on intracellular ROS production, antioxidant defense mechanisms, and the regulation of the PI3K/Akt1 pathway and Runx2 expression. Furthermore, we assessed the potential synergistic effects of the two compounds when used in combination. This study serves as an important preliminary investigation into the potential role of Q in OS therapy.

## 2. Material and Methods

### 2.1. Cell Culture

The human OS cell line MG-63 (ATCC^®^ CRL-1427™, Manassas, VA, USA) was used in this study. Cells were cultured in Dulbecco’s Modified Eagle Medium (DMEM; Gibco™, Thermo Fisher Scientific, Waltham, MA, USA), supplemented with 10% heat-inactivated fetal bovine serum (FBS; Gibco™, Thermo Fisher Scientific) and 1% penicillin-streptomycin solution (100 U/mL penicillin and 100 μg/mL streptomycin; Gibco™, Thermo Fisher Scientific). The cell cultures were maintained in T-75 flasks under standard culture conditions, namely in a humidified atmosphere containing 5% CO_2_ and 95% air at 37 °C. The cells were passaged at approximately 70–80% confluence using 0.25% trypsin-EDTA (Gibco™, Thermo Fisher Scientific), and the medium was refreshed every 2–3 days. All cell culture procedures were performed under aseptic conditions in a certified Class II biosafety cabinet (Labconco, Kansas City, MO, USA). Only cells at passages 3–8 were used in the experiments to ensure phenotypic stability and reproducibility. The duration of the treatment was selected based on the biological endpoint being assessed: 24 h for early-stage apoptosis and ROS measurements, and 48 h for viability, enzymatic activity, and gene expression analyses.

### 2.2. Cell Treatment and Experimental Design

MG-63 osteosarcoma cells were seeded into appropriate culture plates (96-well, black-walled 96-well, or 6-well plates depending on the assay) and allowed to adhere overnight. The cells were then treated with quercetin (Q), doxorubicin (Dox), or their combinations for either 24 or 48 h, depending on the experimental endpoint.

For monotherapy, Q was administered at concentrations of 0, 25, 50, 75, and 100 µM, and Dox at 0, 0.5, 1, 2.5, and 5 µM. Combination treatments were applied at a fixed molar ratio of 5:1 (Q:Dox), including Q25 + Dox5, Q50 + Dox5, and Q100 + Dox5 µM. All treatments were performed in triplicate across at least three independent biological replicates.

Q was dissolved in DMSO to prepare a 100 mM stock solution and Dox was dissolved in sterile distilled water to prepare a 10 mM stock. Working concentrations were freshly prepared in complete DMEM immediately before each experiment. The final DMSO concentration did not exceed 0.1% in any group, including the vehicle control group.

Treatment durations were optimized as follows:

- 24 h: Apoptosis (Annexin V/PI, Caspase-3/7), ROS, gene expression;
- 48 h: Cell viability (MTT), antioxidant enzyme activities, synergy analysis.

### 2.3. Cell Viability Assay and Synergy Analysis

Cell viability was assessed using the MTT assay. MG-63 cells were seeded at a density of 5 × 10^3^ cells per well in 96-well plates and treated as described. After 48 h of treatment, 10 µL of MTT solution (final concentration: 0.5 mg/mL) was added to each well and incubated for 4 h at 37 °C. Formazan crystals were then solubilized with 100 µL DMSO, and absorbance was measured at 570 nm (reference: 630 nm) using a microplate reader (Multiskan GO, Thermo Fisher Scientific). Cell viability was calculated as a percentage relative to the untreated control.

Dose–response curves were generated, and IC_50_ values were calculated using nonlinear regression (variable slope model) in GraphPad Prism v8.0. These IC_50_ values guided the selection of the concentration ranges used in the synergy analyses.

Synergistic interactions between Q and Dox were evaluated using SynergyFinder v2.0 (https://synergyfinder.fimm.fi, accessed on 13 May 2025). A 4 × 4 dose–response matrix combining Q (25, 50, 75, 100 µM) and Dox (1, 2.5, 5, 10 µM) was created based on preliminary optimization. Although the nominal Q:Dox ratio was 5:1, actual ratios ranged from 5:1 to 20:1 to accommodate solubility and viability thresholds. CI values were calculated using the Chou–Talalay method, and synergy was interpreted using the Bliss independence and HSA models. CI < 1 was considered synergistic. The strongest synergism (CI = 0.23) was observed for Q50 + Dox5 µM.

### 2.4. Synergistic Effect Analysis

The analyses were performed using Combenefit software v2.021 (Cancer Research UK Cambridge Institute, Cambridge, UK), a freely available computational tool that generates three-dimensional dose–response interaction surfaces and quantitative synergy metrics. In this study, a fixed molar ratio of 5:1 (Q:Dox) was used for all combination groups, and dose–response matrices were constructed accordingly. These results confirmed the findings obtained from the SynergyFinder analysis presented in Section 2.3.

### 2.5. Apoptosis Analysis

Apoptotic cell death was evaluated using the Annexin V-FITC/Propidium Iodide double staining method and caspase-3/7 enzymatic activity assay. After 48 h of treatment with Q, Dox, or their combination, cells were harvested and washed twice with PBS. Apoptotic staining was performed using the Annexin V-FITC/PI apoptosis detection kit (BioLegend, San Diego, CA, USA) according to the manufacturer’s instructions. Briefly, cells were resuspended in binding buffer, incubated with Annexin V-FITC and PI dyes for 15 min at room temperature in the dark, and immediately analyzed by measuring fluorescence intensity.

In parallel, caspase-3/7 activity was quantified using the Caspase-Glo 3/7 Assay Kit (Promega Corporation, Madison, WI, USA). Equal volumes of cell suspension and assay reagent were mixed in white 96-well plates and incubated for 30 min at room temperature in the dark. Luminescence was measured using a microplate reader, and values were normalized to total protein content determined by a BCA protein assay. All assays were conducted in triplicate, and results were expressed as mean ± standard deviation.

### 2.6. Measurement of Intracellular Reactive Oxygen Species (ROS) Levels

Intracellular ROS levels were measured using the fluorescent probe DCFH-DA (Sigma-Aldrich, St. Louis, MO, USA). Following 48 h of treatment with Q, Dox, or their combination, MG-63 cells were washed twice with PBS to remove residual media and serum components. Cells were then incubated with 10 µM DCFH-DA prepared in serum-free DMEM for 30 min at 37 °C in the dark to prevent photobleaching.

Once internalized, DCFH-DA was deacetylated by intracellular esterases to yield non-fluorescent dichlorodihydrofluorescein (DCFH), which was subsequently oxidized by ROS to form highly fluorescent dichlorofluorescein. After incubation, the cells were washed again with PBS to remove excess dye, and fluorescence intensity was measured using a fluorescence microplate reader (Thermo Fisher Scientific, Waltham, MA, USA) with excitation and emission wavelengths set at 488 nm and 525 nm, respectively.

Each experimental group was run in triplicate, and appropriate controls were included, including untreated control cells and blank wells containing dye but no cells. The fluorescence signal from each well was normalized to total protein content, determined separately using a bicinchoninic acid (BCA) protein assay. ROS levels were expressed as fold change relative to the control group.

### 2.7. Antioxidant Enzyme Activity Assays

To evaluate the cellular antioxidant response following treatment with Q, Dox, or their combination, the enzymatic activities of SOD and GPx were quantified. Analyses were performed using commercially available colorimetric assay kits obtained from Cayman Chemical (Ann Arbor, MI, USA), in accordance with the manufacturer’s instructions.

The SOD assay measured the enzyme’s ability to catalyze the dismutation of superoxide radicals into hydrogen peroxide and molecular oxygen. The colorimetric signal was generated by inhibition of a tetrazolium salt-based reaction and quantified at 450 nm using a microplate reader.

The GPx activity assay was based on a coupled reaction involving the oxidation of NADPH to NADP^+^ during the reduction of hydrogen peroxide to water. The rate of NADPH consumption was monitored by measuring the absorbance decrease at 340 nm.

All experimental groups were run in triplicate, and enzyme activity values were calculated as mean ± standard deviation. These measurements allowed assessment of the oxidative stress status and the antioxidant response induced by each treatment condition.

### 2.8. Cell Lysis and Protein Quantification

Following treatment, MG-63 cells were prepared for enzymatic assays by performing cell lysis under standardized conditions. Cells were washed twice with cold PBS to remove residual medium and detached using a cell scraper. The resulting cell suspensions were transferred to microcentrifuge tubes and lysed using the specific lysis buffer supplied with the antioxidant enzyme assay kits (Cayman Chemical, Ann Arbor, MI, USA).

Cell lysates were incubated on ice for 30 min to ensure complete protein extraction, followed by centrifugation at 10,000× *g* for 15 min at 4 °C to separate cellular debris. The clear supernatants containing soluble proteins were carefully collected and kept on ice until further use.

Total protein concentrations in the lysates were quantified using the BCA protein assay kit (Thermo Fisher Scientific, Waltham, MA, USA) according to the manufacturer’s protocol. Standard curves were generated using bovine serum albumin as the reference protein. Absorbance was measured at 562 nm using a microplate reader, and protein concentrations were calculated accordingly.

All enzyme activity measurements, including those for SOD and GPx, were normalized to total protein content to allow accurate comparisons between experimental groups. Protein quantification and normalization ensured that observed differences in enzyme activity reflected true biological responses rather than variability in sample loading.

### 2.9. Superoxide Dismutase (SOD) Activity

SOD activity was quantified to assess the cellular antioxidant capacity in response to treatment with Q, Dox, or their combination. The assay was performed using a colorimetric SOD assay kit (Cayman Chemical, Ann Arbor, MI, USA), following the manufacturer’s instructions.

The principle of the assay is based on the enzyme’s ability to catalyze the dismutation of superoxide anions (O_2_^−^•) into hydrogen peroxide (H_2_O_2_) and molecular oxygen (O_2_), thereby inhibiting the reduction of a tetrazolium salt substrate. In this assay system, superoxide radicals are generated in vitro and react with a chromogenic tetrazolium salt to form a red formazan dye. In the presence of SOD, the production of superoxide is reduced, leading to a decrease in formazan formation.

After adding the appropriate reagents to the prepared cell lysates, the reaction was initiated and allowed to proceed for the recommended incubation period. Absorbance was measured at 450 nm using a microplate reader. A standard curve was generated using the known concentrations of purified SOD enzyme provided with the kit.

### 2.10. Glutathione Peroxidase (GPx) Activity

GPx activity was determined to evaluate the enzymatic antioxidant response in the MG-63 cells following treatment with Q, Dox, or their combination. The assay was carried out using a commercially available GPx colorimetric assay kit (Cayman Chemical, Ann Arbor, MI, USA) according to the manufacturer’s instructions.

The principle of the assay is based on the ability of GPx to catalyze the reduction of hydrogen peroxide (H_2_O_2_) to water (H_2_O), using reduced glutathione (GSH) as an electron donor. During this reaction, GSH is oxidized to glutathione disulfide (GSSG). To regenerate GSH, glutathione reductase (GR) reduces GSSG back to GSH using NADPH as a cofactor. This coupled reaction allows indirect monitoring of GPx activity through the oxidation of NADPH to NADP^+^.

The decrease in NADPH concentration is measured spectrophotometrically at 340 nm, as NADPH absorbs light at this wavelength, whereas NADP^+^ does not. The rate of decrease in absorbance is directly proportional to GPx enzymatic activity.

### 2.11. RNA Isolation and cDNA Synthesis

Total RNA was isolated from the MG-63 cells using the RNeasy Mini Kit (Qiagen, Hilden, Germany), following the manufacturer’s instructions. Briefly, after removal of the culture medium, the cells were lysed in the supplied lysis buffer containing guanidine–thiocyanate to ensure RNase inactivation and efficient cell disruption. Lysates were homogenized and passed through silica membrane spin columns, followed by a series of washing steps to remove contaminants.

RNA was eluted in RNase-free water and quantified using a NanoDrop 2000 spectrophotometer (Thermo Fisher Scientific, Waltham, MA, USA). The concentration and purity of the RNA were assessed by measuring absorbance at 260 nm and 280 nm, and samples with an A260/A280 ratio between 1.8 and 2.0 were considered acceptable for downstream applications. RNA integrity was further evaluated by electrophoresis on a 1% agarose gel stained with ethidium bromide or a nucleic acid-specific dye, and the presence of intact 28S and 18S rRNA bands was used as an indicator of good quality.

For complementary DNA (cDNA) synthesis, one microgram of total RNA from each sample was reverse transcribed into cDNA using the RevertAid First Strand cDNA Synthesis Kit (Thermo Fisher Scientific, Waltham, MA, USA). The reaction was performed in a final volume of 20 µL using a combination of random hexamer and oligo(dT) primers to ensure comprehensive reverse transcription of both polyadenylated and non-polyadenylated transcripts. The reaction mixture was incubated according to the manufacturer’s thermal protocol (typically 25 °C for 5 min, 42 °C for 60 min, and 70 °C for 5 min for enzyme inactivation). Synthesized cDNA was stored at −20 °C until its use in quantitative real-time PCR (qPCR) analysis.

### 2.12. Real-Time Quantitative PCR Analysis

Quantitative qPCR was performed to assess the mRNA expression levels of PI3K, Akt1, Runx2, and Caspase-3 in MG-63 cells following treatment with Q, Dox, or their combination. The analysis was carried out using SYBR Green chemistry with a commercially available qPCR master mix (Thermo Fisher Scientific, Waltham, MA, USA), following the manufacturer’s protocol.

Amplification reactions were performed in 96-well plates using the CFX96 Touch Real-Time PCR Detection System (Bio-Rad Laboratories, Hercules, CA, USA). Each reaction mixture had a final volume of 20 µL and contained 10 µL of SYBR Green master mix, 1 µL of forward primer, 1 µL of reverse primer, 2 µL of cDNA template, and 6 µL of nuclease-free water. Primer pairs were designed to span exon–exon junctions where possible to prevent amplification of genomic DNA and were validated for specificity and efficiency.

The thermal cycling conditions consisted of an initial denaturation at 95 °C for 3 min, followed by 40 cycles of denaturation at 95 °C for 15 s, annealing at 60 °C for 30 s, and extension at 72 °C for 30 s. A melt curve analysis was conducted at the end of each run to confirm the specificity of the amplification products by gradually increasing the temperature from 65 °C to 95 °C and monitoring fluorescence.

GAPDH was used as the internal reference (housekeeping) gene to normalize the expression levels of target genes. Relative quantification was performed using the comparative Ct method (2^−ΔΔCt^). All reactions were run in triplicate, and each biological condition was tested in three independent experiments. Primer efficiencies were confirmed to fall within the acceptable range of 90% to 110%, and no non-specific amplification or primer dimer formation was observed in the melt curve analysis. The sequences of the primers used in qPCR are provided in Table 1.

### 2.13. Statistical Analysis

Prior to statistical testing, the normality of the data distribution for each dataset was assessed using the Shapiro–Wilk test. This allowed determination of whether parametric or non-parametric statistical methods were appropriate for the subsequent analyses. All quantitative data were obtained from at least three independent biological replicates (n = 3), and each condition included technical replicates to ensure reliability.

The data were expressed as mean values ± standard deviation (SD). For comparison of more than two experimental groups exhibiting non-normally distributed data, the Kruskal–Wallis test was applied as a non-parametric alternative to one-way ANOVA. Where statistically significant differences were detected, post hoc pairwise comparisons were conducted using the Mann–Whitney U test.

A *p*-value of less than 0.05 (*p* < 0.05) was considered statistically significant for all analyses. Graphical representation and statistical calculations were performed using GraphPad Prism software, version 10.0 (GraphPad Software, San Diego, CA, USA). The software was also used to generate dose–response curves, calculate IC_50_ values, and visualize group-wise comparisons in bar plots and scatter plots where appropriate.

## 3. Results

### 3.1. Cell Viability and IC_50_ Values 

According to the MTT assay results, the Dox treatment alone induced a significant, dose-dependent reduction in MG-63 cell viability. As the concentration of Dox increased, cell viability progressively declined to 56%, 50%, 30%, 28%, and 26%, respectively.

**Figure 2:**
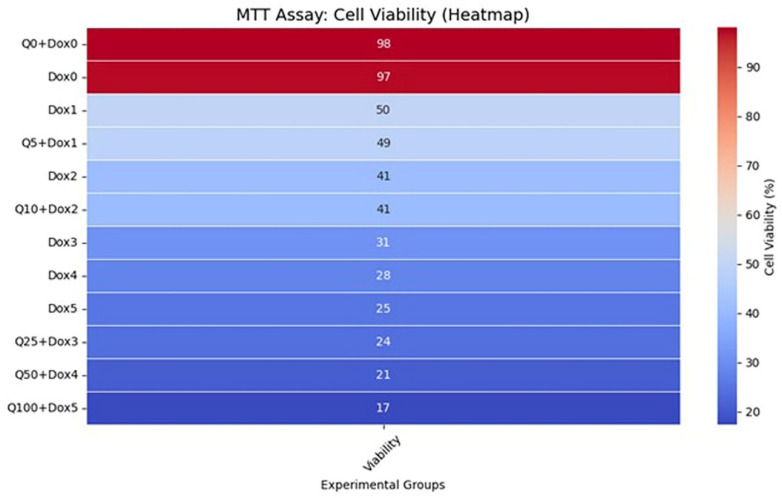
This heatmap illustrates the percentage of viable MG-63 human osteosarcoma cells following 48 h treatments with various concentrations of Dox, Q, and their combinations. Cell viability was quantitatively determined using the MTT assay, which measures mitochondrial metabolic activity as an indirect indicator of cell proliferation and viability. Experimental groups are plotted on the y-axis, while the percentage of viable cells is encoded by a color gradient ranging from blue (low viability) to red (high viability), as indicated by the scale bar on the right. Notably, monotherapy with Dox or Q demonstrated dose-dependent cytotoxicity, whereas the combination treatments—particularly Q100 + Dox5—resulted in the most pronounced decrease in cell viability (17%), indicating a potential synergistic effect between the two agents.

When Q was combined with Dox at a fixed molar ratio of 5:1, a greater cytotoxic effect was observed compared to Dox monotherapy. For instance, while the treatment with Dox at 1 µM resulted in approximately 50% cell viability, this decreased to 48% when combined with Q at 5 µM. Increasing the concentration of the combination further enhanced cytotoxicity. The most pronounced effect was observed in the Dox 5 µM + Q 100 µM group, where cell viability dropped to 17% (Figure 2).

The IC_50_ values were calculated as 70.3 µM for Q and 1.14 µM for Dox. All results were derived from three independent experiments (n = 3). Statistical significance was assessed using the Kruskal–Wallis test followed by pairwise comparisons with the Mann–Whitney U test (* *p* < 0.05, ** *p* < 0.01, *** *p* < 0.001).

**Figure 3:**
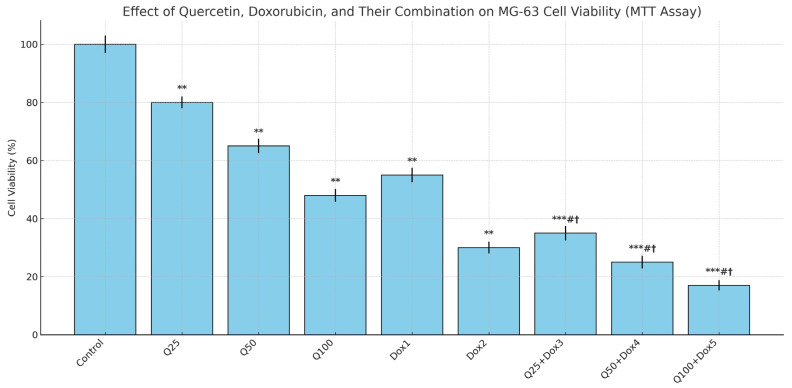
MG-63 cells were treated with increasing concentrations of Q (25, 50, 100 µM), Dox (1, 2 µM), or their combinations (Q25 + Dox3, Q50 + Dox4, Q100 + Dox5) at a fixed molar ratio (5:1) for 48 h. Cell viability was assessed using the MTT assay and is expressed as a percentage of the untreated control group. Bars represent the mean ± standard deviation (SD) from three independent experiments. Statistical analysis was performed using the Kruskal–Wallis test, followed by pairwise comparisons using the Mann–Whitney U test. **, ***: *p* < 0.01, *p* < 0.001 vs. control group. #: *p* < 0.05 vs. quercetin (Q) monotherapy at corresponding concentration. †: *p* < 0.05 vs. doxorubicin (Dox) monotherapy at corresponding concentration.

**Figure 4:**
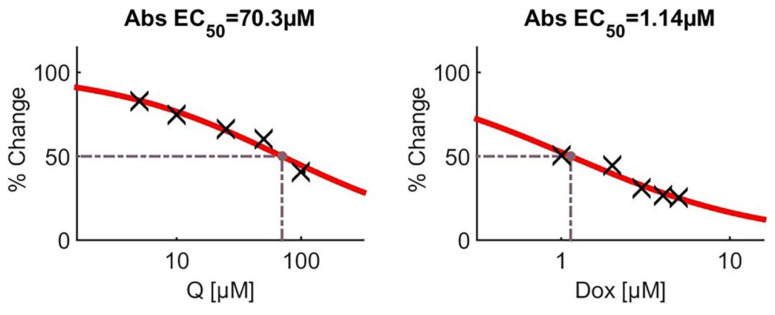
Dose–response curves for Q and Dox monotherapies in MG-63 osteosarcoma cells following 48 h exposure. The curves were generated based on the MTT assay results, and the half-maximal effective concentration (EC_50_) values were calculated using nonlinear regression analysis. For quercetin, the EC_50_ was determined to be 70.3 µM, whereas for doxorubicin it was 1.14 µM, indicating a higher cytotoxic potency of Dox relative to Q. The percent change reflects the reduction in cell viability relative to untreated controls. Black crosses represent individual data points, and the red lines indicate the fitted dose–response model.

Following 48 h of treatment, both Q and Dox significantly inhibited MG-63 cell proliferation in a dose-dependent manner when applied individually (*p* < 0.05). Statistical analysis using the Kruskal–Wallis test revealed significant differences among all the experimental groups (*** *p* < 0.001) (Figure 3). The IC_50_ value for Q was calculated to be approximately 70.3 µM, while that for Dox was 1.14 µM (Figure 4). Furthermore, a more pronounced reduction in cell viability was observed in the combination treatment group compared to either agent alone.

The combination treatment groups (Q25 + Dox3, Q50 + Dox4, Q100 + Dox5) showed significantly lower cell viability compared to both Q and Dox alone, confirming a synergistic cytotoxic effect. While a 5:1 molar ratio of Q to Dox was the intended target in designing the combination treatments, some variations occurred due to practical limitations such as drug solubility and achieving appropriate cytotoxic effects without excessive toxicity. Consequently, the final applied ratios in the combination groups ranged approximately from 5:1 to 20:1. These ratios were selected based on preliminary optimization studies that identified effective concentration ranges capable of inducing synergistic cytotoxicity while maintaining experimental feasibility.

### 3.2. Synergistic Effect Analysis Findings

Synergistic interactions between Q and Dox were evaluated using both the Bliss Independence and HSA models. CI values were interpreted as follows: CI < 1 indicated synergism, CI = 1 indicated an additive effect, and CI > 1 indicated antagonism. All tested combinations yielded CI values below 1, indicating a consistent synergistic interaction between Q and Dox.

**Figure 5:**
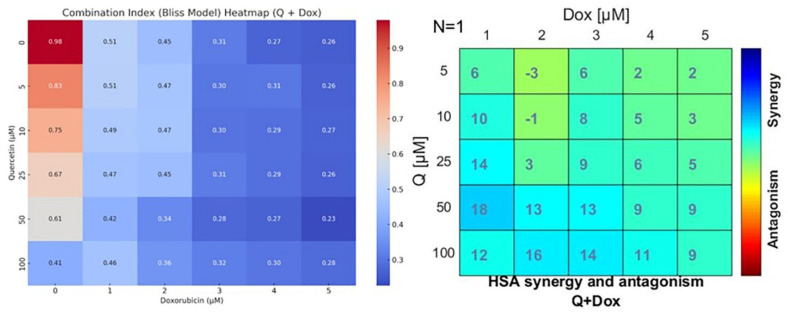
Heatmap representations of drug interaction effects between Q and Dox in MG-63 osteosarcoma cells, as analyzed by two independent synergy models. (**Left panel**) Bliss Independence model-based CI heatmap, where CI values < 1 indicate synergism. The degree of synergism increases as values approach 0. Notably, combination treatments with higher doses of both agents (e.g., Q100 + Dox5) demonstrated strong synergy, as reflected by CI values as low as 0.23. (**Right panel**) HSA model synergy heatmap showing the deviation in percentage effect from the most effective single agent at each concentration. Positive values (blue to cyan) indicate synergy, while negative values (yellow to red) indicate antagonism. The most pronounced synergistic interactions were observed at higher concentrations of quercetin (≥25 µM) combined with Dox (≥3 µM), further supporting the enhanced cytotoxicity of the combinatory approach.

Notably, strong synergy was observed in high-dose combinations, particularly Q50 + Dox5 and Q100 + Dox5, with CI values ranging from 0.22 to 0.27. The most pronounced synergistic effect was detected in the Q50 + Dox5 group (CI = 0.23). These findings were further corroborated by combination response surface plots and heatmaps, which confirmed a dose-dependent synergistic interaction pattern. The synergy plots presented in Figure 5 were generated based on cell viability data from a full 4 × 4 dose–response matrix, encompassing all combinations of Q (25, 50, 75, 100 µM) and Dox (1, 2.5, 5, 10 µM). These data points were derived from the normalized MTT assay results, which served as the input for the SynergyFinder platform. 

Overall, these results suggest that co-treatment with Q and Dox may be an effective strategy to enhance cytotoxicity and reduce cell viability in OS cells (Figure 5; see also Supplementary Figure S1, which provides a full heatmap of cell viability data used for synergy modeling).

It is important to note that the Bliss Independence and HSA synergy models used in this study provide quantitative synergy scores, but do not calculate conventional *p*-values for statistical significance. Therefore, the observed synergy is interpreted based on established thresholds (CI < 1 for synergy in Bliss; positive deviation values in HSA). The reproducibility of these findings was confirmed across three independent experiments. Although the synergy scores (CI values) were derived using the standard Bliss Independence and HSA models via the SynergyFinder 2.0 platform, statistical significance (e.g., *p*-values) was not available from these analyses. Therefore, synergy was interpreted based on predefined model thresholds (CI < 1 for synergy).

### 3.3. Apoptotic Activity Findings

**Figure 6:**
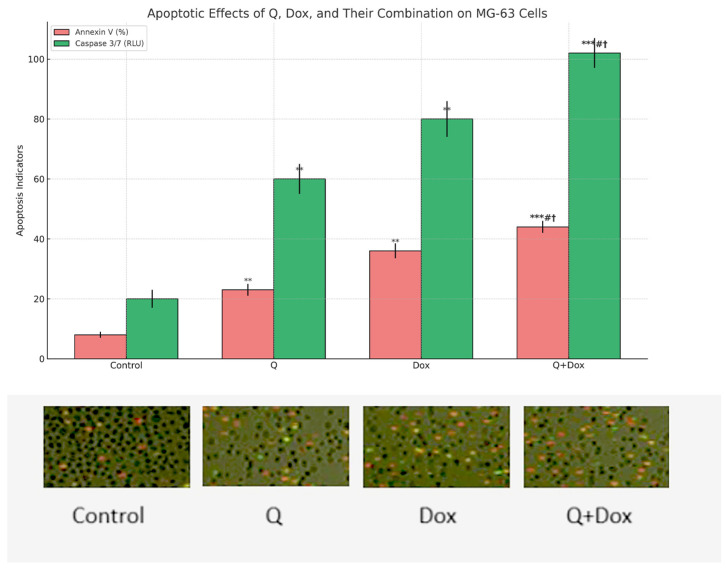
MG-63 cells were treated for 48 h with quercetin (Q, 100 µM), doxorubicin (Dox, 5 µM), or their combination (Q + Dox). Apoptosis was evaluated using Annexin V staining (pink bars, left axis) and caspase 3/7 activity (green bars, right axis). Representative fluorescence microscopy images below graph show Annexin V/PI staining of cells in each treatment group. Bars represent mean ± standard deviation (SD) of three independent experiments. Statistical comparisons were performed using Kruskal–Wallis test followed by Mann–Whitney U test for pairwise analysis. ** and *** denote *p* < 0.01 and *p* < 0.001, respectively, compared to control group. # denotes *p* < 0.05 vs. quercetin monotherapy. † denotes *p* < 0.05 vs. doxorubicin monotherapy.

Apoptosis was assessed by measuring caspase-3/7 enzymatic activity and Annexin V positivity. Caspase-3/7 activity exhibited a progressive increase in all treatment groups compared to the control, with the highest activity observed in the combination group (Q + Dox). Luminescence values were normalized to total protein content to ensure comparability across samples. Statistical analysis confirmed that all treatment conditions significantly increased caspase-3/7 activity relative to the control group (*p* < 0.05) (Figure 6).

Annexin V-FITC/PI staining further supported these findings by demonstrating a marked increase in the proportion of Annexin V-positive cells across all treated groups. The combination treatment yielded the highest apoptotic index, with differences reaching statistical significance compared to the control (*p* < 0.001). All results are expressed as mean ± standard deviation (SD) based on three independent experiments (n = 3).

These results indicate that both Q and Dox independently promote apoptosis in MG-63 cells, and their combined use exerts a synergistic or additive pro-apoptotic effect. The combination treatment group (Q + Dox) exhibited significantly higher levels of apoptotic markers compared to both the Dox and Q monotherapy groups (*p* < 0.05). This indicates that the apoptotic response was not only superior to the control but was also significantly enhanced relative to either agent alone, supporting their synergistic effect.

These results indicate that the combination treatment (Q + Dox) induces significantly higher levels of apoptosis than either treatment alone, supporting a synergistic apoptotic effect.

### 3.4. ROS Level Findings

The Q treatment group exhibited an approximately 30% increase in ROS levels compared to the control (*p* < 0.01), while the Dox group showed a ~44% increase (*p* < 0.01). These results indicate that both agents significantly promote oxidative stress in MG-63 cells. Dox is known to disrupt the mitochondrial electron transport chain, leading to superoxide generation, whereas Q may further augment ROS production through activation of NADPH oxidase.

The combination treatment resulted in a synergistic increase in ROS levels, reaching approximately 60% higher than the control values (*p* < 0.01). This finding suggests that Q enhances the pro-oxidant activity of Dox when they are administered together. While elevated ROS levels may potentiate the cytotoxic effects of treatment by promoting apoptosis in cancer cells, they must be interpreted cautiously due to the potential risk of systemic toxicity, particularly cardiotoxicity.

ROS levels were quantified using the DCFH-DA fluorescent probe and normalized to total protein content (Table 2).

### 3.5. Antioxidant Parameter Findings

Evaluation of antioxidant enzyme activity revealed that Dox treatment induced oxidative stress in MG-63 cells, as evidenced by a 19% reduction in GPx activity and a 15% reduction in SOD activity compared to the control group (*p* < 0.05), indicating impaired antioxidant defense.

**Figure 7:**
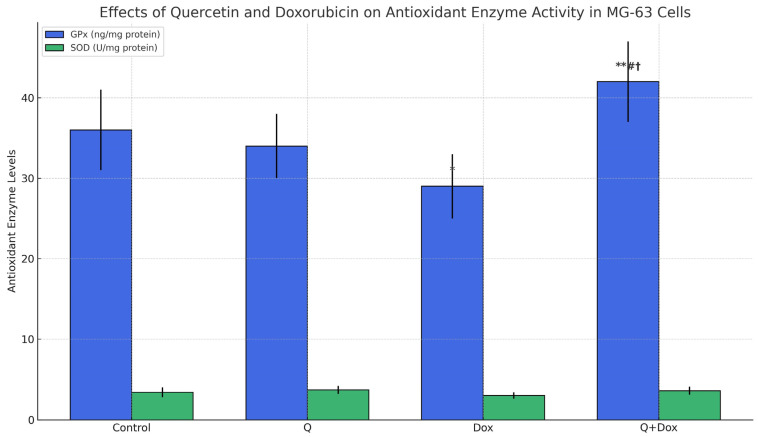
Cells were treated for 48 h, and glutathione peroxidase (GPx; blue bars, left axis) and superoxide dismutase (SOD; green bars, right axis) levels were measured. The doxorubicin group showed a significant reduction in GPx levels compared to the control, while the Q + Dox group significantly restored GPx activity. Bars represent the mean ± standard deviation (SD) from three independent experiments. Statistical comparisons were made using Kruskal–Wallis and Mann–Whitney U tests. * indicates *p* < 0.05 vs. control group. ** indicates *p* < 0.01 vs. control group. # indicates *p* < 0.05 vs. quercetin monotherapy. † indicates *p* < 0.05 vs. doxorubicin monotherapy.

In contrast, treatment with Q alone led to an upregulation of these antioxidant enzymes. Notably, co-treatment with Q and Dox significantly restored the activities of both GPx and SOD compared to the Dox group (*p* < 0.01). This finding suggests that Q may partially compensate for the oxidative damage induced by Dox by enhancing the cellular antioxidant response. The enzyme activity results are presented in Figure 7.

These findings suggest that quercetin mitigates doxorubicin-induced oxidative stress by restoring antioxidant enzyme activity.

### 3.6. Gene Expression Analysis Findings

Runx2 gene expression, which is closely associated with osteogenic differentiation, is known to be highly expressed in OS cells. In the present study, qPCR analysis revealed that Runx2 expression was significantly downregulated in both the Q and Dox treatment groups compared to the control (*p* < 0.05). The most pronounced reduction was observed in the combination group (Q + Dox), suggesting that osteogenic potential may be further suppressed when both agents are administered together.

Akt1, a key gene involved in cell proliferation and survival, was also significantly downregulated following Q and Dox treatment (*p* < 0.01), with the greatest decrease observed in the combination group. This finding implies that co-treatment inhibits the Akt signaling pathway, thereby contributing to reduced proliferation and enhanced apoptosis.

Similarly, PI3K gene expression, which regulates cell growth, proliferation, and survival, was significantly reduced in both single-agent treatments and was further suppressed in the combination group (*p* < 0.01). These results collectively support the conclusion that the PI3K/Akt pathway is a critical target of Q and Dox treatment, and its downregulation is associated with apoptosis induction and cell cycle arrest.

**Figure 8:**
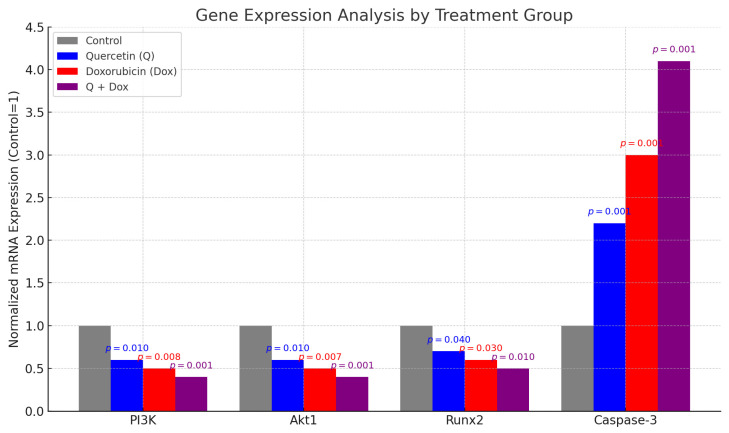
Quantitative analysis of mRNA expression levels of PI3K, Akt1, Runx2, and Caspase-3 genes in MG-63 osteosarcoma cells following treatment with Q, Dox, or their combination (Q + Dox). Gene expression levels were normalized to the control group (set to 1) and presented as mean ± standard deviation (SD). PI3K and Akt1: A significant downregulation was observed in all treatment groups compared to the control (*p* < 0.05), with the most pronounced suppression detected in the Q + Dox combination group (*p* = 0.001), suggesting inhibition of the PI3K/Akt signaling pathway. Runx2: mRNA expression was significantly reduced following the Q, Dox, and especially Q + Dox treatments (*p* = 0.040, *p* = 0.030, and *p* = 0.010, respectively), indicating possible suppression of osteogenic transcriptional activity. Caspase-3: In contrast, a marked upregulation of Caspase-3 gene expression was observed in all treatment groups, with the combination therapy producing the highest induction (approximately 4-fold increase; *p* = 0.001), supporting enhanced apoptotic activation.

Conversely, expression of the pro-apoptotic effector caspase-3 was significantly upregulated in all treatment groups, with the highest level observed in the Q + Dox group (*p* < 0.001). This confirms that combination therapy robustly activates the apoptotic machinery in MG-63 cells (Figure 8).

The qPCR results were normalized to GAPDH as the internal reference gene. Melt curve analysis confirmed amplification specificity, and the primer efficiencies were within the acceptable range of 90–110%. RNA integrity was validated via agarose gel electrophoresis.

The relative mRNA expression levels of the Runx2, Akt1, PI3K, and Caspase-3 genes in the MG-63 cells after treatment with Q, Dox, and their combination (Q + Dox) are presented in the table below. Compared to the control group, both Q and Dox significantly downregulated the expression of Runx2, PI3K, and Akt1, with the most substantial reductions observed in the combination group (Q + Dox). Conversely, Caspase-3 expression was significantly upregulated, particularly in the Q + Dox group, indicating a strong induction of apoptosis. GAPDH was used as the internal reference gene. These findings support the conclusion that co-treatment with Q and Dox modulates both proliferative and apoptotic gene expression pathways in a synergistic manner (Table 3).

## 4. Discussion

This study provides comprehensive evidence of the apoptotic and antioxidative effects of Q, both alone and in combination with Dox, in MG-63 OS cells. Q, a flavonoid with well-documented anticancer and antioxidant properties, was evaluated for its potential to enhance Dox’s chemotherapeutic efficacy while simultaneously mitigating its oxidative side effects. OS, the most prevalent malignant bone tumor in adolescents, remains a therapeutic challenge due to its frequent chemoresistance and treatment-associated toxicity. The present findings demonstrate that both Q and Dox significantly suppressed cell proliferation and induced apoptosis in MG-63 cells, with their combined treatment showing markedly superior efficacy compared to monotherapy.

Molecular analyses revealed a significant increase in Caspase-3 gene expression in the treatment groups, particularly in the combination group, indicating activation of intrinsic mitochondrial apoptotic pathways. Concurrently, a marked decrease in PI3K, Akt1, and Runx2 gene expression was observed, supporting the conclusion that Q effectively inhibits proliferative and survival pathways while also suppressing the osteogenic characteristics of OS cells. MTT assays confirmed that Dox decreased cell viability in a dose-dependent manner, and this effect was significantly enhanced when combined with Q. Specifically, the combination of 5 µM Dox and 100 µM Q reduced cell viability by up to 16 percent. The IC_50_ values also support this observation, with Dox exhibiting an IC_50_ of 1.14 µM, whereas Q had an IC_50_ of approximately 70.3 µM. These results indicate that although Dox is effective even at low concentrations, its cytotoxic efficacy is significantly amplified when combined with Q [12]. Similar synergistic interactions have been documented in previous cancer studies involving Q and other chemotherapeutic agents [13,14].

Synergy analyses using the Bliss Independence and HSA models demonstrated CI values below 1 in all combination groups, indicating synergistic interactions. The most pronounced synergy was observed in the group treated with 50 μM Q and 5 μM Dox, where the CI value was calculated as 0.23. This calculation was performed using the Chou–Talalay method implemented in SynergyFinder, which models drug interactions based on the median-effect principle [15,16,17]. This finding is consistent with prior reports highlighting the synergistic effects of Q in combination with established chemotherapeutic drugs.

Apoptotic induction was further validated through biochemical assays that showed increased Caspase-3/7 activity and Annexin V positivity in the combination group. Caspase-3 mRNA expression was also significantly elevated in this group, providing additional evidence for enhanced activation of apoptotic mechanisms. These results align with previously published studies indicating the pro-apoptotic potential of Q in various cancer cell types [18,19].

ROS levels increased significantly following treatment with either Dox or Q, with the combination treatment inducing the highest ROS accumulation, reaching approximately 60 percent. This suggests that oxidative stress contributes to the observed apoptotic activity and may underlie the enhanced efficacy of the combination. Although increased ROS levels are associated with cytotoxic effects, the antioxidant capacity of Q may also serve to mitigate the systemic side effects of chemotherapy, such as Dox-induced cardiotoxicity [20].

Dox treatment significantly decreased the activities of antioxidant enzymes including GPx and SOD. However, these enzyme levels were restored or even elevated with Q treatment, especially in the combination group. This finding supports the role of Q in reducing oxidative damage and preserving cellular antioxidant defense mechanisms [21]. The ability of Q to simultaneously promote cancer cell death and protect against oxidative injury positions it as a promising candidate for adjuvant therapy.

From a mechanistic standpoint, inhibition of the PI3K/Akt pathway has been linked to G1/S phase cell cycle arrest and the promotion of apoptosis in OS cells [22]. In the present study, both Q and Dox suppressed this pathway, accompanied by an increased expression of Caspase-3, indicating enhanced apoptotic signaling [23,24]. The observed downregulation of Runx2, a transcription factor involved in osteogenic differentiation, further suggests a reduction in the tumorigenic phenotype. In parallel, suppression of Akt1 indicates attenuation of pro-survival signaling, contributing to reduced cell proliferation and increased apoptotic sensitivity.

Our data are in agreement with previous findings that highlight the pivotal role of p53 in mediating mitochondrial apoptosis in response to genotoxic agents. Goloudina et al. demonstrated that chemotherapeutic-induced stress activates p53, leading to mitochondrial outer membrane permeabilization and caspase activation independently from the death receptor pathway. In our study, Dox treatment led to a marked upregulation of p53 gene expression, coupled with increased caspase-3 activity, supporting the notion that intrinsic apoptosis is the predominant mechanism. These results strengthen the hypothesis that p53 acts as a molecular integrator linking DNA damage to mitochondrial apoptotic signaling in osteosarcoma cells [25].

Despite the promising findings presented in this study, several limitations should be acknowledged. First, the experimental design was confined to a single osteosarcoma cell line, MG63, which may limit the generalizability of the results across other osteosarcoma subtypes with distinct genetic and phenotypic profiles. Second, although the combination of quercetin and doxorubicin demonstrated synergistic effects in vitro, the absence of in vivo validation restricts the translational applicability of the findings, particularly in terms of bioavailability, pharmacokinetics, and systemic toxicity. Third, while key apoptotic markers and signaling molecules such as PI3K, Akt1, Runx2, and Caspase 3 were evaluated at the messenger RNA level, the corresponding protein expression levels and posttranslational modifications were not assessed. This limits the depth of the mechanistic insight into the pathways involved. The current study utilized basic in vitro assays to evaluate cytotoxicity, apoptosis, oxidative stress, and gene expression. Although these methods are widely accepted for initial screening, they do not reflect the full biological complexity of osteosarcoma. The effects of quercetin and doxorubicin were assessed at two time points, with twenty-four hours used for early responses such as apoptosis and reactive oxygen species generation, and forty-eight hours for viability, antioxidant enzyme activity, and gene expression. However, long-term outcomes such as clonogenic survival, recurrence, and drug resistance were not investigated. Additionally, while a range of dose combinations were tested, the main focus was placed on a fixed molar ratio of five to one, which may not fully represent the optimal combination. Off-target effects and systemic toxicity were also not evaluated in this study. Addressing these limitations in future studies including in vivo models, protein-level validation, extended treatment durations, and the use of multiple cell lines will be essential to fully elucidate the therapeutic potential and clinical relevance of quercetin in osteosarcoma treatment.

Building on the current findings, future research should focus on validating the synergistic anticancer effects of Q and Dox in vivo using animal models of OS. This would allow for a comprehensive evaluation of pharmacokinetics, biodistribution, and systemic toxicity, particularly in relation to Dox-induced cardiotoxicity and the antioxidant-protective potential of Q. In addition, studies employing other OS cell lines and patient-derived tumor models would help confirm the generalizability of the observed effects. Although the present study primarily focused on Dox concentrations near its IC_50_, the HSA model suggested that synergy may also occur at lower Dox levels. Therefore, future in vitro and in vivo studies should investigate sub-IC_50_ Dox doses in combination with Q to further optimize therapeutic efficacy while minimizing potential toxicity. Advanced molecular techniques, including Western blotting and proteomic analysis, should be utilized to assess protein-level changes in apoptotic and survival pathways, thus deepening mechanistic understanding. Investigation into the role of other signaling pathways potentially modulated by Q, such as NF-κB and MAPK, may also provide valuable insight. Furthermore, formulation strategies aimed at improving the bioavailability and tumor-targeting efficiency of Q, including nanoparticle-based delivery systems, represent a promising area for future development. Collectively, these efforts may pave the way for the clinical translation of Q as an adjuvant to standard chemotherapy in OS.

## 5. Conclusions

In conclusion, this study demonstrates that the combined application of Q and Dox exerts a synergistic antitumor effect on MG-63 OS cells. Q enhances the cytotoxicity of Dox by promoting intrinsic apoptosis, inhibiting oncogenic signaling pathways, and modulating redox balance. Its dual role in both increasing apoptotic responses and reducing oxidative damage supports its potential as an adjuvant in OS chemotherapy. However, further validation in in vivo models and with additional OS cell lines, as well as comprehensive assessments of systemic toxicity, will be essential to translate these findings into clinical practice. These findings provide a compelling rationale for the inclusion of Q in combined chemotherapeutic strategies against OS and warrant further preclinical and clinical investigation.

## Figures and Tables

**Table 1:** Primer sequences used (5′–3′ direction).

Gene Name	Forward Primer	Reverse Primer
*Runx2*	5′- CCGCCTCAGTGATTTAGGGC -3′	5′- GGGTCTGTAATCTGACTCTGTCC -3′
*Akt1*	5′- CCTTCATTGGCTACGACGTG -3′	5′- GTCAGCCCTGCTTCTCTGAG -3′
*PI3K (p110α)*	5′- ACGAGCAGGGAGACTTTGAA -3′	5′- TCCACAGTAGCCAAAGTCCAG -3′
*Caspase-3*	5′- GGAAGCGAATCAATGGACTCT -3′	5′- CGCAAAGTGACTGGATGAACC -3′
*GAPDH*	5′- GAAGGTGAAGGTCGGAGTCA -3′	5′- GAAGATGGTGATGGGATTTC -3′

**Table 2:** ROS production in treatment groups (* *p* < 0.01).

Treatment Group	Mean Fluorescence Intensity (a.u.)	*p*-Value vs. Control	Significance
Control	100.3 ± 3.1	-	-
Q	129.7 ± 2.8	<0.01	*
Dox	144.5 ± 4.2	<0.01	*
Q + Dox	160.1 ± 3.5	<0.01	*

* *p* < 0.01 indicates a statistically significant difference compared to the control group (Kruskal–Wallis test followed by Mann–Whitney U test).

**Table 3:** qPCR gene expression results. (*** *p* < 0.001, ** *p* < 0.01, * *p* < 0.05).

Gene	Control (Mean ± SD)	Q (Mean ± SD)	Dox (Mean ± SD)	Q + Dox (Mean ± SD)	*p*-Value
Runx2	1.00 ± 0.05	0.62 ± 0.04	0.51 ± 0.03	0.29 ± 0.02	<0.05 *
Akt1	1.00 ± 0.06	0.59 ± 0.05	0.48 ± 0.04	0.22 ± 0.03	<0.01 **
PI3K	1.00 ± 0.04	0.61 ± 0.06	0.53 ± 0.05	0.25 ± 0.02	<0.01 **
Caspase-3	1.00 ± 0.03	1.78 ± 0.08	1.96 ± 0.06	2.85 ± 0.07	<0.001 ***

## Data Availability

All datasets generated during the current study are available from the corresponding author upon reasonable request. Raw data and original figures can be provided for verification purposes.

## Supplementary Materials

The following supporting information can be downloaded at https://www.mdpi.com/article/10.3390/medicina61081347/s1, Figure S1. Heatmap of MG-63 Cell Viability (%) Across Q + Dox Combination Matrix.

## Abbreviations

The following abbreviations are used in this manuscript:

Q	Quercetin
Dox	Doxorubicin
OS	Osteosarcoma
Runx2	Runt-related transcription factor 2
ROS	Reactive oxygen species
PI3K/Akt1	Phosphoinositide 3-kinase/protein kinase B
CI	Combination index
RT-qPCR	Reverse transcription quantitative polymerase chain reaction
PARP	Poly (ADP-ribose) polymerase
DMSO	Dimethyl sulfoxide
HSA	Highest single agent
PBS	Phosphate-buffered saline
GPx	Glutathione peroxidase
SOD	Superoxide dismutase
DCFH-DA	2′,7′-dichlorofluorescin diacetate
BCA	Bicinchoninic acid
GSH	Reduced glutathione
cDNA	Complementary DNA
qPCR	Quantitative real-time PCR
