# Supplementary figures and images for: PI3K/Akt1 Pathway Suppression by Quercetin–Doxorubicin Combination in Osteosarcoma Cell Line (MG-63 Cells)
Source: Medicina (Kaunas). 2025 Jul 25;61(8):1347. doi: 10.3390/medicina61081347 (PMC12387616; doi:10.3390/medicina61081347)

Supplementary Figure S1. Heatmap of MG-63 Cell Viability (%) Across Q + Dox Combination Matrix

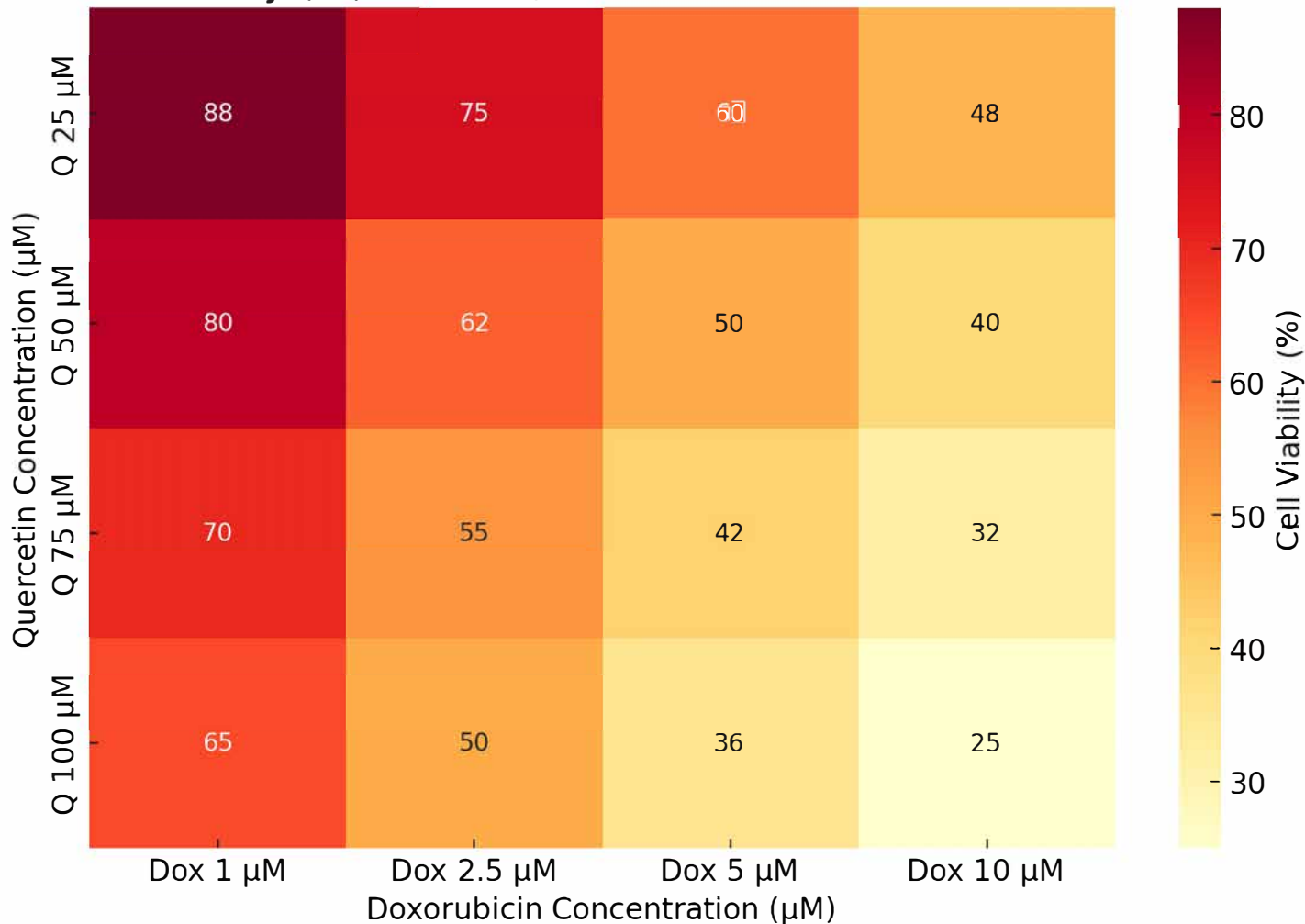

Supplement: Supplementary file 1 [file medicina-61-01347-s001.zip › medicina-3727522-supplementary.pdf]
